# Supplementary material for: RP-UHPLC-MS Chemical Profiling, Biological and In Silico Docking Studies to Unravel the Therapeutic Potential of Heliotropium crispum Desf. as a Novel Source of Neuroprotective Bioactive Compounds
Source: Biomolecules. 2021 Jan 4;11(1):53. doi: 10.3390/biom11010053 (PMC7824284; doi:10.3390/biom11010053)
Supplement: Supplementary file 1 [file biomolecules-11-00053-s001.pdf]

biomolecules

Article

RP-UHPLC-MS Chemical Profiling, Biological and *In Silico* Docking Studies to Unravel the Therapeutic Potential of *Heliotropium Crispum* Desf. as a Novel Source of Neuroprotective Bioactive Compounds

Adeel Arshad[1], Saeed Ahemad[1], Hammad Saleem[2,3], Muhammad Saleem[4], Gokhan Zengin[5], Hassan H. Abdallah[6], Muhammad Imran Tousif[7], Nafees Ahemad[2] and Mohamad Fawzi Mahomoodally[8,9]*

[1]*Department of Pharmacy, Faculty of Pharmacy and Alternative Medicine, The Islamia University of Bahawalpur, 63100, Pakistan; adeelarshad.11@gmail.com; rsahmed_iub@yahoo.com*

[2]*School of Pharmacy, Monash University Malaysia, Jalan Lagoon Selatan, 47500 Bandar Sunway, Selangor Darul Ehsan, Malaysia; nafees.ahemad@monash.edu; hammad.saleem@monash.edu;hammad.saleem@uvas.edu.pk*

[3]*Institute of Pharmaceutical Sciences (IPS), University of Veterinary & Animal Sciences (UVAS), Lahore-54000, Pakistan; hammad.saleem@uvas.edu.pk*

[4]*Department of Chemistry, The Islamia University of Bahawalpur, Bahawalpur-63100, Pakistan; m.saleem@iub.edu.pk*

[5]*Selcuk University, Science Faculty, Department of Biology, Konya-Turkey; gokhanzengin@selcuk.edu.tr*

[6]*Chemistry Department, College of Education, Salahaddin university-Erbil, 44001, Erbil, Iraq; Hassan.abdullah@su.edu.krd*

[7]*Department of Chemistry, Township Campus, University of Education Lahore, Pakistan; imran.tousif@ue.edu.pk*

[8]*Department for Management of Science and Technology Development, Ton Duc Thang University, Ho Chi Minh City, Vietnam*

[9]*Faculty of Applied Sciences, Ton Duc Thang University, Ho Chi Minh City, Vietnam*

* Correspondence: *mohamad.fawzi.mahomoodally@tdtu.edu.vn (M.F.M)*

SUPPLEMENTARY MATERIAL

1. Phytochemical composition

1.1. Total phenolic contents

The total phenolic content was determined as previously described in the literature [1] with some modifications. Sample solution (1 mg/mL; 0.25 mL) was mixed with diluted Folin–Ciocalteu reagent (1 mL, 1:9, v/v) and shaken vigorously. After 3 min, Na_2CO_3 solution (0.75 mL, 1%) was added and the sample absorbance was read at 760 nm after 2 h incubation at room temperature [2].

1.2. Total flavonoid contents

The total flavonoids content was determined using $AlCl_3$ method [3]. Briefly, sample solution (1 mg/mL; 1 mL) was mixed with the same volume of aluminum trichloride (2%) in methanol. Similarly, a blank was prepared by adding a sample solution (1 mL) to methanol (1 mL) without $AlCl_3$. The sample and blank absorbances were read at 415 nm after 10 min incubation at room temperature. The absorbance of the blank was subtracted from that of the sample [4].

1.3. UHPLC-MS secondary metabolites analysis

UHPLC Accurate-Mass Q-TOF (Agilent 1290 Infinity LC system coupled to Agilent 6520) mass spectrometer with dual ESI source was used. Column specifications were as: XDB-C18 Agilent Zorbax Eclipse, narrow-bore 2.1 x 150 mm, 3.5 micron (P/N: 930990-902). The temperature of the column was maintained at 25 °C, while the auto-sampler temperature was 4 °C. The following two mobile phases used were: A (0.1% formic acid in water), B (0.1% formic acid in acetonitrile) at a flow rate of 0.5 mL/min. Injection volume was 1.0 μL. Run time was 25 min and post-run time was 5 min. MS analysis full scan was carried out over a range of *m/z* 100-1000 employing electrospray ion source in the negative ionization mode. The flow rate for nitrogen as nebulizing and drying gas was 25 and 600 L/hour, respectively with the drying gas temperature of 350 °C. The fragmentation voltage was optimized to 125. Capillary voltage for analysis was 3500 V [5].

2. Antioxidant assays

2.1. DPPH

For the DPPH (1,1-diphenyl-2-picrylhydrazyl) radical scavenging assay: Sample solution (1 mg/mL; 1 mL) was added to 4 mL of a 0.004% methanol solution of DPPH. The sample absorbance was read at 517 nm after a 30 min incubation at room temperature in the dark. DPPH radical scavenging activity was expressed as millimoles of trolox equivalents (mg TE/g extract) [3,6].

2.2. ABTS

For ABTS (2,20-azino-bis(3-ethylbenzothiazoline) 6-sulfonic acid) radical scavenging assay: Briefly, ABTS+ was produced directly by reacting 7 mM ABTS solution with 2.45 mM
potassium persulfate and allowing the mixture to stand for 12–16 in the dark at room temperature. Prior to beginning the assay, ABTS solution was diluted with methanol to an absorbance of 0.700 ± 0.02 at 734 nm. Sample solution (1 mg/mL; 1 mL)
was added to ABTS solution (2 mL) and mixed. The sample absorbance was read at 734 nm after a 30 min incubation at room temperature [3,6]. The ABTS radical scavenging activity was expressed as millimoles of trolox equivalents (mmol TE/g extract) [7].

2.3. FRAP

For FRAP (ferric reducing antioxidant power) activity assay: Sample solution (1 mg/mL; 0.1 mL) was added to premixed FRAP reagent (2 mL) containing acetate buffer (0.3 M, pH 3.6), 2,4,6-tris(2-pyridyl)-*S*-triazine (TPTZ) (10 mM) in 40 mM HCl and ferric chloride (20 mM) in a ratio of 10:1:1 (v/v/v). Then, the sample absorbance was read at 593 nm after a 30 min incubation at room temperature. FRAP activity was expressed as milligrams of trolox equivalents (mg TE/g extract) [3,6].

2.4. CUPRAC

For CUPRAC (cupric ion reducing activity) activity assay: Sample solution (1 mg/mL; 0.5 mL) was added to premixed reaction mixture containing $CuCl_2$ (1 mL, 10 mM), neocuproine (1 mL, 7.5 mM) and NH_4Ac buffer (1 mL, 1 M, pH 7.0). Similarly, a blank was prepared by adding sample solution (0.5 mL) to premixed reaction mixture (3 mL) without CuCl2. Then, the sample and blank absorbances were read at 450 nm after a 30 min incubation at room temperature. The absorbance of the blank was subtracted from that of the sample. CUPRAC activity was expressed as milligrams of trolox equivalents (mg TE/g extract) [3,6].

2.5. Phosphomolybdenum

For phosphomolybdenum method: Sample solution (1 mg/mL; 0.3 mL) was combined with 3 mL of reagent solution (0.6 M sulfuric acid, 28 mM sodium phosphate and 4 mM ammonium molybdate). The sample absorbance was read at 695 nm after a 90 min incubation at 95°C. The total antioxidant capacity was expressed as millimoles of trolox equivalents (mmol TE/g extract) [8].

2.6. Metal chelating

For metal chelating activity assay: Briefly, sample solution (1 mg/mL; 2 mL) was added to FeCl2 solution (0.05 mL, 2 mM). The reaction was initiated by the addition of 5 mM ferrozine (0.2 mL). Similarly, a blank was prepared by adding sample solution (2 mL) to FeCl2 solution (0.05 mL, 2 mM) and water (0.2 mL) without ferrozine. Then, the sample and blank absorbances were read at 562 nm after 10 min incubation at room temperature. The absorbance of the blank was subtracted from that of the sample. The metal chelating activity was expressed as milligram [3,6].

3. Enzyme inhibition assays

3.1. Cholinesterases

For AChE inhibitory activity assay: Sample solution (1 mg/mL; 50 μL) was mixed with DTNB (5,5-dithio-bis(2-nitrobenzoic) acid, Sigma, St. Louis, MO, United States) (125 μL) and AChE [acetylcholines-terase (Electric ell AChE, Type-VI-S, EC 3.1.1.7, Sigma)], or BChE [BChE (horse serum BChE, EC 3.1.1.8, Sigma)] solution (25 μL) in Tris–HCl buffer (pH 8.0) in a 96-well microplate and incubated for 15 min at 25°C. The reaction was then initiated with the addition of acetylthiocholine iodide (ATCI, Sigma) or butyrylthiocholine chloride (BTCl, Sigma) (25 μL). Similarly, a blank was prepared by adding sample solution to all reaction reagents without enzyme (AChE or BChE) solution. The sample and blank absorbances were read at 405 nm after 10 min incubation at 25°C. The absorbance of the blank was subtracted from that of the sample and the cholinesterase inhibitory activity was expressed as galanthamine equivalents (mg GALAE/g extract) [9].

3.2. Amylase and glucosidase

For α-amylase inhibitory activity assay: Sample solution (1 mg/mL; 25 μL) was mixed with α-amylase solution (ex-porcine pancreas, EC 3.2.1.1, Sigma) (50 μL) in phosphate buffer (pH 6.9 with 6 mM

sodium chloride) in a 96-well microplate and incubated for 10 min at 37◦C. After pre-incubation, the reaction was initiated with the addition of starch solution (50 μL, 0.05%). Similarly, a blank was prepared by adding sample solution to all reaction reagents without enzyme (*α*-amylase) solution. The reaction mixture was incubated 10 min at 37◦C. The reaction was then stopped with the addition of HCl (25 μL, 1 M). This was followed by addition of the iodine-potassium iodide solution (100 μL). The sample and blank absorbances were read at 630 nm. The absorbance of the blank was subtracted from that of the sample and the *α*-amylase inhibitory activity was expressed as acarbose equivalents (mmol ACE/g extract) [10].

For *α*-glucosidase inhibitory activity assay: Sample solution (1 mg/mL; 50 μL) was mixed with glutathione (50 μL), *α*-glucosidase solution (from Saccharomyces cerevisiae, EC 3.2.1.20, Sigma) (50 μL) in phosphate buffer (pH 6.8) and PNPG (4-*N*-trophenyl-*α*-D-glucopyranoside, Sigma) (50 μL) in a 96-well microplate and incubated for 15 min at 37◦C. Similarly, a blank was prepared by adding sample solution to all reaction reagents without enzyme (*α*-glucosidase) solution. The reaction was then stopped with the addition of sodium carbonate (50 μL, 0.2 M). The sample and blank absorbances were read at 400 nm. The absorbance of the blank was subtracted from that of the sample and the *α*-glucosidase inhibitory activity was expressed as acarbose equivalents (mmol ACE/g extract) [11].

3.3. Tyrosinase

For tyrosinase inhibitory activity assay: Sample solution (1 mg/mL; 25 μL) was mixed with tyrosinase solution (40 μL, Sigma) and phosphate buffer (100 μL, pH 6.8) in a 96-well microplate and incubated for 15 min at 25◦C. The reaction was then initiated with the addition of L-DOPA (40 μL, Sigma). Similarly, a blank was prepared by adding sample solution to all reaction reagents without enzyme (tyrosinase) solution. The sample and blank absorbances were read at 492 nm after a 10 min incubation at 25◦C. The absorbance of the blank was subtracted from that of the sample and the tyrosinase inhibitory activity was expressed as kojic acid equivalents (mg KAE/g extract) [12].

References

1. Slinkard, K.; Singleton, V.L. Total phenol analysis: automation and comparison with manual methods. *American journal of enology and viticulture* **1977**, *28*, 49-55.
2. Vlase, L.; Mocan, A.; Hanganu, D.; Benedec, D.; Gheldiu, A.; Crisan, G. Comparative study of polyphenolic content, antioxidant and antimicrobial activity of four Galium species (Rubiaceae). *Digest Journal of Nanomaterials and Biostructures* **2014**, *9*, 1085-1094.
3. Zengin, G.; Uysal, A.; Gunes, E.; Aktumsek, A. Survey of phytochemical composition and biological effects of three extracts from a wild plant (Cotoneaster nummularia Fisch. et Mey.): a potential source for functional food ingredients and drug formulations. *PLoS One* **2014**, *9*, e113527.
4. Uysal, S.; Zengin, G.; Locatelli, M.; Bahadori, M.B.; Mocan, A.; Bellagamba, G.; De Luca, E.; Mollica, A.; Aktumsek, A. Cytotoxic and enzyme inhibitory potential of two Potentilla species (P. speciosa L. and P. reptans Willd.) and their chemical composition. *Frontiers in pharmacology* **2017**, *8*, 290.
5. Saleem, H.; Htar, T.T.; Naidu, R.; Nawawi, N.S.; Ahmad, I.; Ashraf, M.; Ahemad, N. Biological, chemical and toxicological perspectives on aerial and roots of Filago germanica (L.) huds: Functional approaches for novel phyto-pharmaceuticals. *Food and Chemical Toxicology* **2019**, *123*, 363-373.

6. Dezsi, Ș.; Bădărău, A.S.; Bischin, C.; Vodnar, D.C.; Silaghi-Dumitrescu, R.; Gheldiu, A.-M.; Mocan, A.; Vlase, L. Antimicrobial and antioxidant activities and phenolic profile of Eucalyptus globulus Labill. and Corymbia ficifolia (F. Muell.) KD Hill & LAS Johnson leaves. *Molecules* **2015**, *20*, 4720-4734.
7. Mocan, A.; Schafberg, M.; Crișan, G.; Rohn, S. Determination of lignans and phenolic components of Schisandra chinensis (Turcz.) Baill. using HPLC-ESI-ToF-MS and HPLC-online TEAC: Contribution of individual components to overall antioxidant activity and comparison with traditional antioxidant assays. *Journal of Functional Foods* **2016**, *24*, 579-594.
8. Mocan, A.; Zengin, G.; Uysal, A.; Gunes, E.; Mollica, A.; Degirmenci, N.S.; Alpsoy, L.; Aktumsek, A. Biological and chemical insights of Morina persica L.: a source of bioactive compounds with multifunctional properties. *Journal of Functional Foods* **2016**, *25*, 94-109.
9. Mocan, A.; Zengin, G.; Crişan, G.; Mollica, A. Enzymatic assays and molecular modeling studies of Schisandra chinensis lignans and phenolics from fruit and leaf extracts. *Journal of enzyme inhibition and medicinal chemistry* **2016**, *31*, 200-210.
10. Savran, A.; Zengin, G.; Aktumsek, A.; Mocan, A.; Glamoćlija, J.; Ćirić, A.; Soković, M. Phenolic compounds and biological effects of edible Rumex scutatus and Pseudosempervivum sempervivum: potential sources of natural agents with health benefits. *Food & function* **2016**, *7*, 3252-3262.
11. Llorent-Martínez, E.; Ortega-Barrales, P.; Zengin, G.; Uysal, S.; Ceylan, R.; Guler, G.; Mocan, A.; Aktumsek, A. Lathyrus aureus and Lathyrus pratensis: characterization of phytochemical profiles by liquid chromatography-mass spectrometry, and evaluation of their enzyme inhibitory and antioxidant activities. *RSC Advances* **2016**, *6*, 88996-89006.
12. Mocan, A.; Zengin, G.; Simirgiotis, M.; Schafberg, M.; Mollica, A.; Vodnar, D.C.; Crişan, G.; Rohn, S. Functional constituents of wild and cultivated Goji (L. barbarum L.) leaves: phytochemical characterization, biological profile, and computational studies. *Journal of enzyme inhibition and medicinal chemistry* **2017**, *32*, 153-168.

Publisher's Note: MDPI stays neutral with regard to jurisdictional claims in published maps and institutional affiliations.

© 2021 by the authors. Submitted for possible open access publication under the terms and conditions of the Creative Commons Attribution (CC BY) license (http://creativecommons.org/licenses/by/4.0/).
